# The ADRP domain from a virulent strain of infectious bronchitis virus is not sufficient to confer a pathogenic phenotype to the attenuated Beaudette strain

**DOI:** 10.1099/jgv.0.001098

**Published:** 2018-06-12

**Authors:** Sarah Keep, Erica Bickerton, Maria Armesto, Paul Britton

**Affiliations:** The Pirbright Institute, Surrey, UK

**Keywords:** infectious bronchitis virus, coronavirus, ADRP, macrodomain, nsp3, pathogenicity

## Abstract

The replicase gene of the coronavirus infectious bronchitis virus (IBV) encodes 15 non-structural proteins (nsps). Nsp 3 is a multi-functional protein containing a conserved ADP-ribose-1″-phosphatase (ADRP) domain. The crystal structures of the domain from two strains of IBV, M41 (virulent) and Beaudette (avirulent), identified a key difference; M41 contains a conserved triple-glycine motif, whilst Beaudette contains a glycine-to-serine mutation that is predicted to abolish ADRP activity. Although ADRP activity has not been formally demonstrated for IBV nsp 3, Beaudette fails to bind ADP-ribose. The role of ADRP in virulence was investigated by generating rIBVs, based on Beaudette, containing either a restored triple-glycine motif or the complete M41 ADRP domain. Replication *in vitro* was unaffected by the ADRP modifications and the *in vivo* phenotype of the rIBVs was found to be apathogenic, indicating that restoration of the triple-glycine motif is not sufficient to restore virulence to the apathogenic Beaudette strain.

## Full-Text

Infectious bronchitis virus (IBV) is a gammacoronavirus that is responsible for an acute highly contagious and economically important respiratory disease, infectious bronchitis, in domestic fowl. IBV possesses a large (27.5 kb) single-stranded positive-sense RNA genome. The 5′-end encompassing approximately two-thirds of the genome encodes 15 non-structural proteins (nsps), which collectively are commonly referred to as the replicase gene. The 3′ third of the genome encodes the structural and accessory genes in the following order; spike (S), accessory genes 3a, 3b, envelope (E), membrane (M), accessory genes 4b, 5a, 5b and nucleocapsid (N). Previous research has investigated the role of the IBV structural and accessory genes, as well as the replicase gene, in pathogenicity, with the latter demonstrated to be a pathogenic determinant [1].

The largest nsp within the coronavirus replicase gene repertoire is nsp 3, which is a multi-functional protein containing a number of putative domains that are found to be conserved amongst different coronaviruses (reviewed in [2]). One such domain is the X domain, also known as the macrodomain or ADRP domain due to its ADP-ribose-1″- phosphatase activity [3–6]. The binding of ADP-ribose and poly(ADP)ribose, and the subsequent catalytic action, have been well characterized in several coronaviruses, with a number of essential residues identified. Work by several groups involving the mutation of these residues in the betacoronaviruses SARS-CoV and MHV, as well as the alphacoronavirus HCoV-229E, demonstrated that ADRP activity is not required for viral replication *in vitro* [5, 7, 8].

Recently, alternative functions for the ADRP domain [9–11], and its potential targets [12], have been proposed. For example, Fehr *et al*. believe that the domain may act as a de-MAR/PARylating enzyme [9]. ADP-ribosylation is a post-translational modification in which one (mono) or more (poly) ADP-ribose moieties are attached to a protein, and this process can be used as an infection signal. Li *et al.* [11] demonstrated that viral macrodomains can reverse this modification, and that residues that are essential for ADP-ribose-1″-phosphate phosphatase activity are also essential for de-MAR/PARylating activities [10]. Despite the exact function of the ADRP domain remaining unknown, several groups have characterized the *in vivo* phenotype associated with an inactive ADRP domain. Eriksson *et al.* demonstrated that an MHV with an inactive ADRP domain, as a result of the mutation asparagine-1348 to alanine (N1348A), did not cause acute viral hepatitis in mice [7]. Reduced interleukin 6 (IL-6) production was observed in the spleen and liver, indicating that an active ADRP domain enhances MHV-induced liver pathology through the induction of inflammatory cytokines. Kuri *et al*. examined the effect of the equivalent mutation (N1040A) in SARS-CoV and HCoV-229E (N1305A) [8]. Recombinant viruses with inactivated ADRP domains were found to have an increased sensitivity to interferon alpha (IFN-α). Fehr *et al*. modified a mouse-adapted SARS-CoV ADRP domain and demonstrated that an inactive ADRP domain resulted in reduced viral load in the lungs of infected mice, accompanied by reduced pathology [9]. Increases in the levels of IFN-α, IFN-β, interferon-stimulated genes and proinflammatory cytokines were observed. Overall, this has resulted in the suggestion that the inactivation of ADRP led to the control of viral replication, thus reducing the viral load and subsequently affecting the clinical outcome. Although there may be differing methods for the action of ADRP between the coronaviruses, there is mounting evidence that the ADRP domain plays a role in virulence and the regulation of innate immune responses to infection [7–10].

The determination of the crystal structure of the ADRP protein from several coronaviruses, including SARS-CoV, HCoV-229E and IBV, [13, 14] has provided a wealth of information, particularly with regard to key residues in the binding site, and those involved in catalytic activity. One such set of residues, found to be largely conserved between coronaviruses, is the triple-glycine (Gly-Gly-Gly) motif that lines the ADP-ribose-binding cleft [14]. The structure of the IBV ADRP protein has been determined from two strains, the apathogenic strain Beaudette [14] and the pathogenic strain M41 [13]. Interestingly, the second glycine, residue 1051, in the Beaudette ADRP is replaced with serine residue, resulting in the replacement of the conserved triple-glycine motif with Gly-Ser-Gly. Piotrowski *et al.* [14] demonstrated that the replacement of Beaudette G1051S resulted in a structural change within ADRP, when compared to the M41 ADRP structure, which distorts the ADP-ribose-binding cleft by displacing a phenylalanine at position 1136. Egloff *et al*. [3] identified that Gly-48, corresponding to residue 1051 in IBV Beaudette, was one of two essential amino acids for SARS-CoV ADRP activity. Binding studies using isothermal titration calorimetry and zone-interference gel electrophoresis have shown that the Beaudette ADRP protein fails to bind ADP-ribose or poly(ADP) ribose [14]. The pathogenic IBV strain M41 does not have the serine mutation within the ADP-ribose-binding cleft [13], and is therefore assumed to have the ability to bind ADP-ribose.

The determination of the crystal structures of the IBV ADRP proteins from the avirulent Beaudette and virulent M41 strains and the identification of this key difference within the binding cleft raised an interesting hypothesis with regard to pathogenicity. The loss of the triple-glycine motif and subsequent inactivation of the Beaudette ADRP opened up the opportunity to use our reverse genetics system to determine whether the loss of ADRP activity was responsible for or involved in the attenuation of the IBV Beaudette. In this study, we investigated whether the inability of Beaudette to bind ADP-ribose is responsible for attenuation and the resulting loss of pathogenicity. Using our reverse genetics system we modified the Beaudette ADRP domain by (1) replacing the serine residue at position 1051 with glycine to restore the triple-glycine motif and the potential to bind ADP-ribose and thus restore ADRP activity, and (2) replacing the entire Beaudette ADRP sequence, amino acid residues 1003 to 1171, with the corresponding sequence from the virulent M41 strain to rule out other potential attenuating mutations present in the Beaudette ADRP domain. The IBV nucleotide and amino acid positions are based on the Beaudette-CK sequence (GenBank accession number AJ311317).

**Fig. 1.**
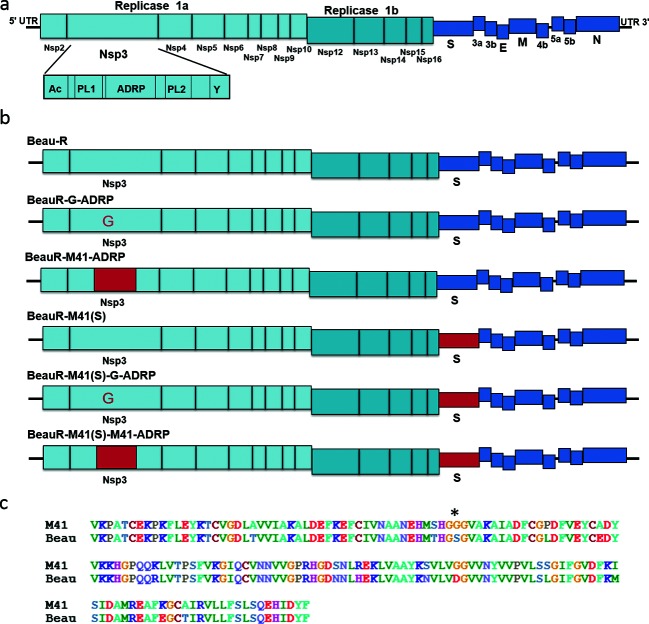
The ADRP domain. (a) Schematic of the IBV genome. The ADRP domain is located within non-structural protein (nsp) 3, encoded within the replicase gene. Nsp 3 is the largest of the nsps, and consists of several domains, including transmembrane domains, an acidic hypervariable region (Ac), papain-like cysteine proteinases (PL1 and PL2) and the Y region. (b) Schematic representation of the rIBVs analysed in this study. The sequence derived from M41 is shown in red and the sequence derived from Beau-R is shown in blue. (c) Sequence alignment of the ADRP domain from M41 and Beaudette, amino acid residues 1003–1171 of the IBV replicase protein. The ADRP domain is largely conserved. An asterisk (*) highlights the difference within the binding cleft.

An A to G point mutation, resulting in an amino acid change from serine to glycine (S1051G) in the Beaudette ADRP, was introduced into a cDNA copy of the Beaudette (Beau-R) genome within a recombinant vaccinia virus [15]. In addition, we also introduced the same change into the IBV cDNA of BeauR-M41(S) [16, 17], which consists of the Beaudette genome but with the S gene from M41. BeauR-M41(S) has the potential to infect host cells *in vivo* due to the presence of the M41 S gene and was included in our investigations to rule out the possibility that the Beaudette S protein may affect infection *in vivo* and therefore the restoration of a virulent phenotype. Recombinant IBVs (rIBVs) BeauR-G-ADRP and BeauR-M41(S)-G-ADRP (Fig. 1b) were successfully rescued and propagated in primary chicken kidney (CK) cells.

Sequence alignment of the ADRP domains from Beau-R and M41-CK identified 36 nucleotide differences resulting in 14 amino acid changes, including the serine/glycine substitution at position 1051 (Fig. 1c). To rule out other possible attenuating mutations within the Beaudette ADRP domain, the coding sequence of amino acid residues 1003–1171 of the IBV replicase protein, representing the complete IBV ADRP domain, were replaced with the corresponding M41 nucleotide sequence in both Beau-R and BeauR-M41(S), generating rIBVs BeauR-M41-ADRP and BeauR-M41(S)-M41-ADRP (Fig. 1b). Both rIBVs were rescued and propagated in CK cells. All rIBVs were passaged four times in CK cells, stocks were prepared and sequence analysis showed no reversions or modifications to the modified ADRP sequences within the recombinant viruses.

**Fig. 2.**
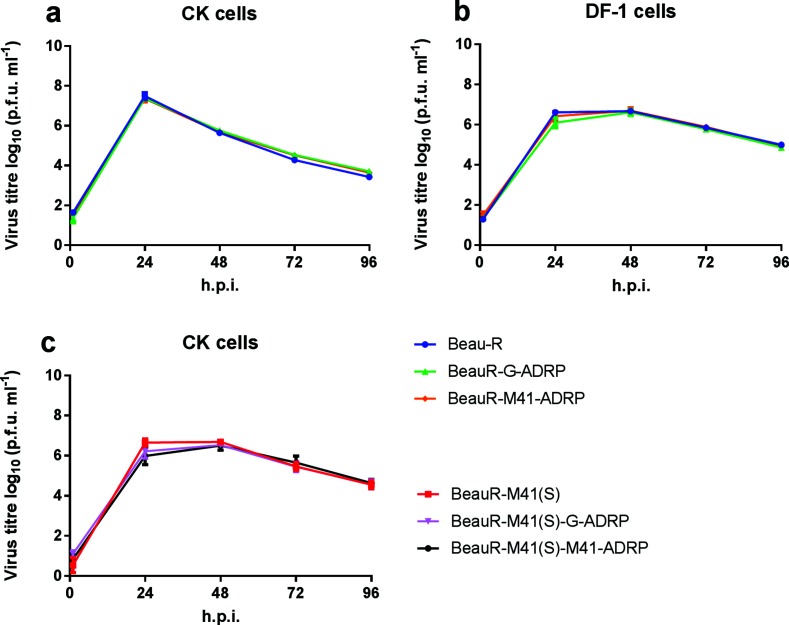
Activation of ADRP has no effect on viral replication *in vitro*. Primary chicken kidney (CK) cells and DF-1 cells, a continuous cell line derived from chicken embryo fibroblasts, were inoculated with 5×10^4^ plaque-forming units (p.f.u.) of (a) rIBV BeauR-G-ADRP, Beau-R or BeauR-M41-ADRP, or (b) rIBV BeauR-M41(S), BeauR-M41(S)-G-ADRP or BeauR-M41(S)-M41-ADRP. Supernatant, containing viral progeny, was harvested at 1, 24, 48, 72 and 96 h post-infection (h.p.i.), and was titrated in triplicate on CK cells. The means of three independent experiments are plotted, with the error bars representing the standard error of the mean. The data were analysed using a one-way analysis of variance (ANOVA) with a Friedman test and Dunn’s multiple comparison test; the replication of the rIBVs was not statistically different to that of the parent viruses, rIBV Beau-R and rIBV BeauR-M41(S).

The growth kinetics of all four rIBVs were determined using primary CK cells, and were found to be comparable to those of the parental viruses, Beau-R and BeauR-M41(S) (Fig. 2). The Beaudette S gene confers extended *in vitro* cell tropism [17]. To evaluate whether the ADRP modification effects could be cell-specific, the growth kinetics were also investigated in DF-1 cells, a continuous cell line derived from chicken embryo fibroblasts; no difference in replication kinetics were observed. In addition, the plaque morphology for all rIBVs was comparable to that of the parental viruses in both DF-1 and CK cells (data not shown). The restoration of the triple-glycine motif thought to activate ADRP in the Beaudette replicase either by a complete domain swap or by an amino acid substitution had no observable effect on viral replication *in vitro*.

**Fig. 3.**
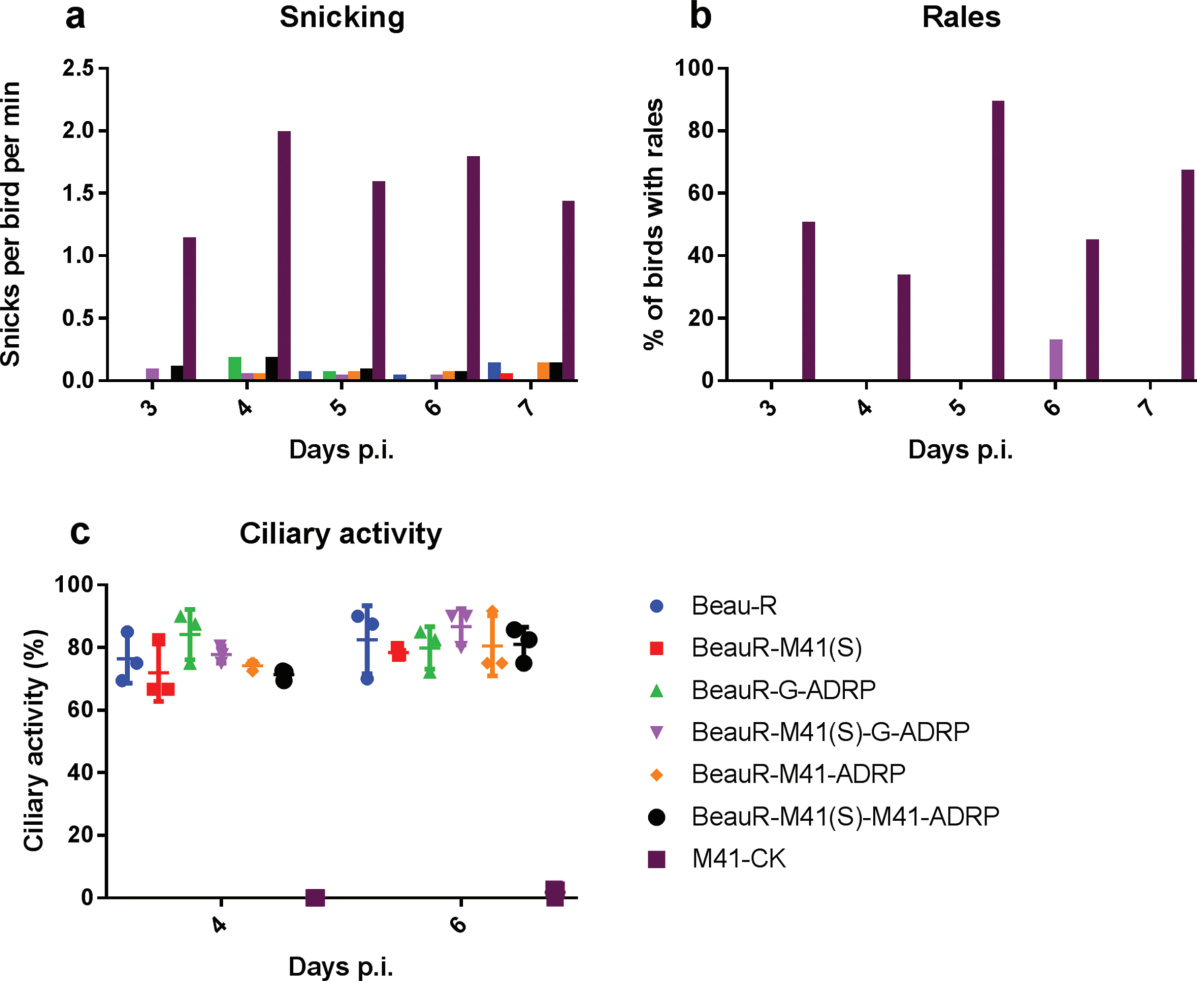
An active ADRP domain is insufficient to confer pathogenicity to rIBV Beau-R. Eight-day-old chickens were inoculated with 10^4^ p.f.u. of either M41-CK, Beau-R, BeauR-M41(S), BeauR-G-ADRP, BeauR-M41-ADRP, BeauR-M41(S)-G-ADRP or BeauR-M41(S)-M41-ADRP. Clinical signs, such as (a) snicking and (b) rales, were assessed on days 3 to 7 post-infection. Birds infected with BeauR-G-ADRP displayed no clinical signs, similarly to those infected with Beau-R. In contrast, snicking and rales were observed in chickens infected with M41-CK from day 3 post-inoculation. (c) Four and 6 days post-infection, three birds per group were culled and the ciliary activity in 10×1 mm trachea sections was assessed by light microscopy and the percentage ciliary activity was calculated. The retention of less than 50 % tracheal ciliary activity 4 days post-infection is indicative of the presence of a pathogenic isolate of IBV. The error bars represent the standard error of the mean. Ciliary activity was analysed using a one-way ANOVA with a Friedman test and Dunn’s multiple comparison test. The ciliary activity was statistically comparable between Beau-R- and BeauR-G-ADRP-infected chickens, confirming that rIBV BeauR-G-ADRP is avirulent in chickens.

In order to determine whether the restoration of the triple-glycine motif to the Beaudette ADRP had any effect on the restoration of pathogenicity, individually housed groups of specific pathogen-free (SPF) Rhode Island Red (RIR) chickens were inoculated via the intra-ocular and intra-nasal routes with 10^4^ plaque-forming units (p.f.u.) of either rIBV BeauR-G-ADRP, BeauR-M41-ADRP, BeauR-M41(S)-G-ADRP or BeauR-M41(S)-M41-ADRP, or parental control viruses, Beau-R, BeauR-M41(S) or M41-CK. The chickens were observed for clinical signs, snicking, watery eyes, nasal discharge and rales, associated with a pathogenic IBV, from days 3 to 7 post-infection (Fig. 3a, b). Clinical signs were not observed in chickens infected with control parental rIBVs Beau-R or BeauR-M41(S), as previously observed [16], whereas chickens infected with M41-CK displayed clinical signs that peaked 4 days post-infection. Chickens infected with the rIBVs BeauR-G-ADRP or BeauR-M41-ADRP exhibited no clinical signs, comparable to the Beau-R-infected group. Similarly, birds infected with rIBVs BeauR-M41(S)-G-ADRP or BeauR-M41(S)-M41-ADRP displayed no clinical signs, comparable to BeauR-M41(S), suggesting that even with altered tropism, an active ADRP domain expressed in a Beaudette genome is not sufficient to confer a pathogenic *in vivo* phenotype. The avirulent phenotype of the rIBVs was further confirmed following the assessment of ciliary activity in tracheal rings produced from tracheas extracted from chickens 4 and 6 days post-infection. Loss of ciliary activity is used as a marker for the presence of IBV in the trachea, with a ciliary activity score of 100 % indicating that no virus is present. Conversely, a reduced ciliary activity score of between 0–50 %, with 0 % termed ciliostasis, indicates the presence of IBV actively replicating and destroying tracheal epithelial cells, which is indicative of a pathogenic isolate. The chickens infected with M41-CK exhibited a significant reduction in ciliary activity in comparison to the parental control Beau-R and BeauR-M41(S) groups (Fig. 3c). Analysis of tracheal organ rings at 4 days post-infection from the chickens infected with the rIBVs based on Beaudette but with modified ADRP domains, BeauR-G-ADRP, BeauR-M41-ADRP, BeauR-M41(S)-G-ADRP and BeauR-M41(S)-M41-ADRP, showed average (mean) ciliary activities of 84, 74, 78 and 71 %, respectively, comparable to those from the groups infected with Beau-R (76 %) and BeauR-M41(S) (78 %). A similar pattern was also observed on day 6 post-infection. To further confirm the presence or absence of IBV in the trachea, RNA was extracted from tracheal cells on days 4 and 6 post-infection and analysed by IBV real-time RT-PCR following a previously described protocol [18]. IBV RNA was only detected in tracheal cells isolated from M41-CK-infected chickens (data not shown).

The absence of IBV RNA in the tracheal cells isolated from chickens infected with the ADRP rIBVs, alongside comparable ciliary activities and clinical signs to Beau-R- and BeauR-M41(S)-infected chickens, demonstrates that neither the ADRP domain from M41, nor the serine to glycine repair is enough to restore pathogenicity to the apathogenic rIBVs Beau-R or BeauR-M41(S). The loss of virulence associated with the Beaudette replicase gene therefore is likely due to a more dominant mutation or set of mutations. The ability of the rIBV Beau-R, as well as the parent virus, IBV Beau-CK, both containing the glycine-to-serine mutation in the binding cleft, to replicate efficiently in continuous and primary cells supports the notion that ADRP function is non-essential *in vitro*. This is further supported by the fact that the replacement of the ADRP domain in Beau-R with its M41 counterpart, or the serine-to-glycine mutation, has not resulted in increased *in vitro* viral replication. These observations are in agreement with previous research into the inactivation of the ADRP in other coronaviruses, which also did not appear to have an effect on RNA replication or the production of viable virus progeny *in vitro*. Seemingly in contrast to previous research in which the inactivation of the ADRP domain resulted in attenuation, the restoration of the triple-glycine motif to the Beaudette ADRP domain in this study did not restore virulence to Beaudette. This indicates that there is a dominant attenuating mutation or set of mutations still present within the virus genome. Our results involving the restoration of the triple-glycine motif to the Beaudette ADRP domain with no observable increase in virulence do not ultimately rule out its involvement in loss of virulence.
